# Perceived discrepancies in neurosonology training and certification across Europe: a RRFS/EAN survey

**DOI:** 10.3389/fneur.2024.1464946

**Published:** 2024-10-28

**Authors:** Vlad Tiu, João Durães, Francesco Di Lorenzo, Nina Vashchenko, Alicia Gonzalez-Martinez, Alice Accorroni, Vanessa Carvalho, Giacomo Sferruzza, Luca Cuffaro

**Affiliations:** ^1^Stroke Unit, Elias University Emergency Hospital, Bucharest, Romania; ^2^Neurology Department, Carol Davila University of Medicine and Pharmacy, Bucharest, Romania; ^3^Neurology Department, Hospitais da Universidade de Coimbra, ULS, Coimbra, Portugal; ^4^Faculty of Medicine, University of Coimbra, Coimbra, Portugal; ^5^Foundation Santa Lucia, Rome, Italy; ^6^Department of Neurology, Danish Headache Center, Copenhagen University Hospital – Rigshospitalet, Copenhagen, Denmark; ^7^University Headache Clinic, Moscow, Russia; ^8^Hospital Universitario de la Princesa, Madrid, Spain; ^9^Instituto de Investigación Sanitaria Princesa (IIS-Princesa), Madrid, Spain; ^10^Geneva Memory Center, Department of Rehabilitation and Geriatrics, Geneva University Hospitals, Geneva, Switzerland; ^11^Department of Neurosciences and Mental Health, Hospital de Santa Maria, Centro Hospitalar Universitário Lisboa-Norte, Lisbon, Portugal; ^12^Centro de Estudos Egas Moniz, Faculdade de Medicina, Universidade de Lisboa, Lisbon, Portugal; ^13^Neurology Unit, IRCCS Ospedale San Raffaele Scientific Institute, Milan, Italy; ^14^Vita-Salute San Raffaele University, Milan, Italy; ^15^School of Medicine and Surgery, University of Milano-Bicocca, Milan, Italy

**Keywords:** neurology, neurosonology, ultrasonogrophy, neuro-pocus, residency, curricula, training, survey

## Abstract

**Introduction:**

Neurosonology is a vital paraclinical investigation in modern neurology. However, access to education and certification in neurosonology for neurology residents and young specialists in Europe is challenging, and comprehensive data regarding this topic are scarce. Information regarding difficulties in neurosonology training across Europe may help bring this topic under the spotlight and act as a call for the harmonization of curricula across the continent.

**Methods:**

We performed an online survey targeting European neurology residents and young specialists, focusing on neurosonology training and certification. The survey was conducted between May and September 2023 and received responses from 282 participants representing 37 European countries.

**Results:**

There were disparities in neurosonology training during residency, with 6 (16.2%) out of 37 countries reporting a dedicated curriculum. The respondents expressed an overall lack of satisfaction with theoretical knowledge (rating their experience as very poor 28.0%, poor 20.2%, neutral 25.9%, good 19.3%, and very good 6.6%) and practical skills gained during their training (rating their experience as very poor 30.9%, poor 18.9%, neutral 22.6%, good 18.1%, and very good 9.5%). A total of 282 respondents (5.7%), 16 held a national certification in neurosonology, claiming obstacles such as high costs of certification and a limited number of certifying centers.

**Discussion:**

This survey reveals significant variations in neurosonology training across Europe, indicating difficulties in obtaining certification. Despite the increasing importance of neurosonology, many neurologists feel inadequately prepared and lack practical training during residency, emphasizing the need for better and more standardized access.

**Conclusion:**

The survey underscores challenges and disparities in neurosonology training and certification in Europe. Standardization of curricula and increased awareness about available certifications are crucial to address these issues. The interest in European Certification suggests a potential solution for enhancing neurosonology training at the international level.

## Introduction

Neurosonology is a rapidly evolving field that uses ultrasound technology to provide vital diagnostic information in the assessment and monitoring of neurovascular pathologies (1). Its non-invasive nature, bedside availability, and real-time capabilities offer unique advantages over other imaging modalities (2). Applications of neurosonology include, but are not limited to, the following:

Cerebrovascular disease: Assessing carotid and vertebral artery stenosis, detecting intracranial occlusions, and monitoring vasospasms in conditions such as subarachnoid hemorrhage (1).

Brain parenchyma evaluation: Identifying midline shifts associated with space-occupying lesions and visualizing brain structures in certain clinical conditions (3).

Neuromuscular ultrasound (NMUS): Evaluating nerve and muscle pathology, such as carpal tunnel syndrome or peripheral neuropathy (4).

Peri-procedural monitoring and guidance: During interventions such as carotid artery stenting or thrombolysis (5).

Point-of-care ultrasound in the neuro-intensive care unit (neuro-ICU) setting: Neurosonology can be a valuable tool in the neuro-ICU for a variety of purposes, such as the assessment of midline shift, intracranial hemorrhage, hydrocephalus, vasospasm, intracranial pressure, cerebral circulatory arrest, and ultrasound-guided lumbar puncture (6).

Due to its invaluable role in neurological diagnostics, neurosonology is often included in neurology residency training curricula but not for all European countries (7). Standardized training pathways and accessible certification in neurosonology remain limited for residents and young neurologists (defined within EAN/RRFS as resident/physician in training in Neurology, Research Fellow in Neurology, PhD student in Neurology, all three groups up to a maximum of 3 years beyond their latest degree) across Europe. Variations exist in access to formal education and the acquisition of necessary skills (8). Furthermore, different medical specialties may have primary responsibility for performing neurosonology examinations, including neurologists, radiologists, and vascular surgeons, leading to further disparities between countries (9, 10).

Expanding access to neurosonology training and certification is crucial to ensuring optimal patient care. While some countries have national-level certifications or society-based programs, a wider, standardized approach is needed. This article will explore the current landscape of neurosonology training and certification across Europe, highlighting existing barriers and variations.

## Methods

### Study design and participants

We designed a joint online survey on education in neurosonology, dementia, and pain. The survey targeted residents and young specialists in Neurology from Europe (i.e., all 48 countries accepted as full members within the EAN), who were contacted via email or accessed through their national representative of the Resident and Research Fellow Section (RRFS) of the European Academy of Neurology (EAN). The survey was accessible online from May 2023 and closed in September 2023.

### Eligibility criteria

Individuals doing their training in Neurology (or that had performed most of their training) in Europe were eligible for the study and up to 3 years after finishing their training.

### Study variables and protocol

The section on neurosonology consisted of 19 questions related to experience in training and certification in neurosonology. The survey is included as Supplementary material.

### Outcome measures

The primary objective was to evaluate the presence of dedicated curricula for Neurosonology across neurology training in Europe. Secondary objectives included the description of the type of training, duration, and curricula distribution as well as the main challenges faced during skill acquisition across different training programs in Europe.

### Statistical analysis

The data collected were processed using Microsoft Excel (Microsoft Corp., Seattle, United States) and IBM SPSS (SPSS Inc., Chicago, IL, United States). Demographic and baseline characteristics were described using medians and interquartile ranges or means and standard deviations for continuous variables, depending on the normality of the distribution, and percentages for categorical data. We evaluated the normality of the distribution using Kolmogorov–Smirnov and Levene’s tests.

## Results

There were a total of 282 responders. Of these, 96 were junior neurologists/consultants (34.0%), 141 (50.0%) were residents, and 12 were post-doctoral students (4.3%). There were 56 (19.9%) respondents who were PhD students, with 33 of them being also involved in residency programs or working as junior neurologists, and 23 of them being exclusively involved in their PhD studies.

**Figure 1:**
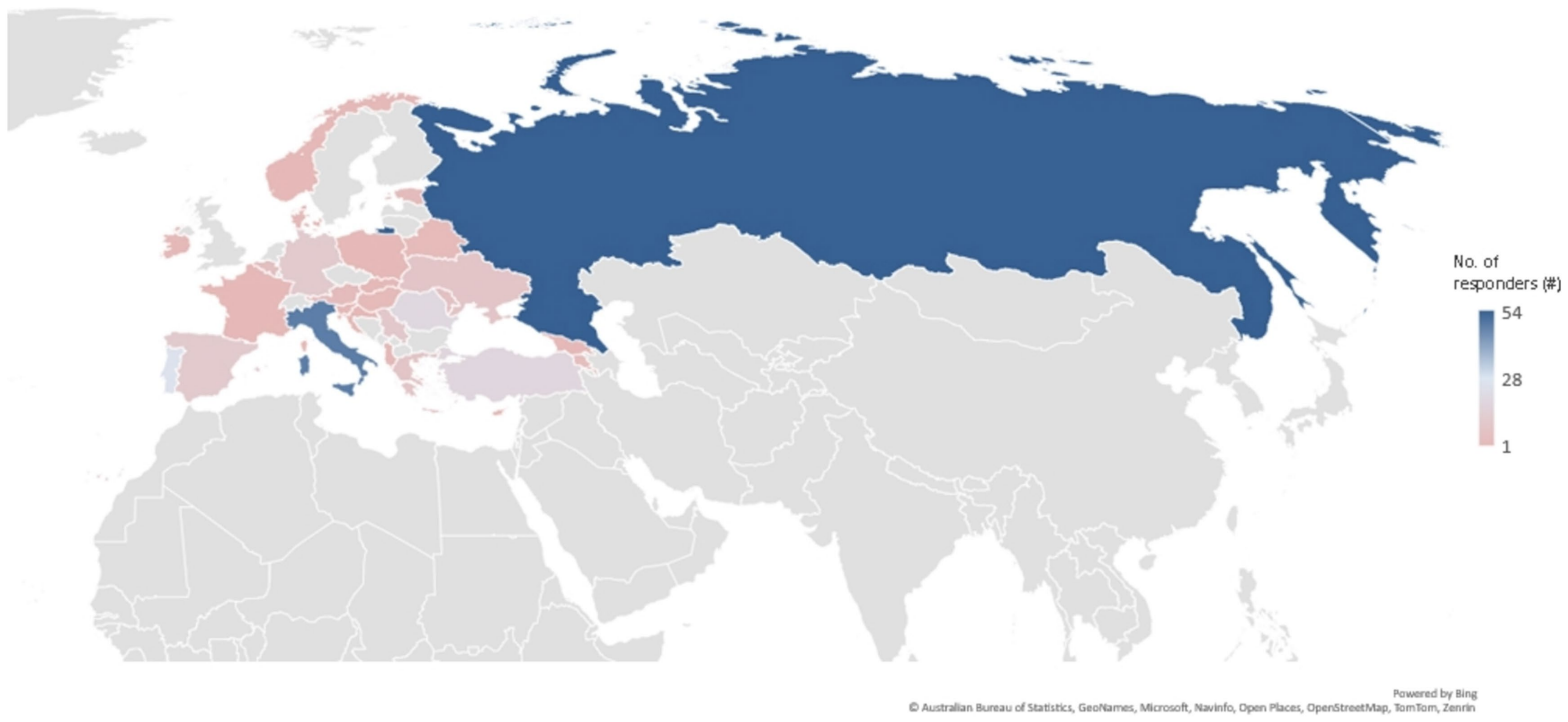
Distribution of responders across countries.

A total of 37 European countries (as accepted within EAN) were represented (Albania, Armenia, Austria, Azerbaijan, Belarus, Belgium, Croatia, Cyprus, Denmark, Estonia, France, Georgia, Germany, Greece, Hungary, Ireland, Italy, Kazakhstan, Lithuania, Moldova, Montenegro, the Netherlands, North Macedonia, Norway, Poland, Portugal, Romania, Russia, Serbia, Slovakia, Slovenia, Spain, Switzerland, Turkey, Ukraine, the United Kingdom, and Uzbekistan). The distribution of respondents by country was uneven, with 58.2% of respondents coming from five respondent countries (Russia—54 (19.1%), Italy—48 (17%), Portugal—25 (8.9%), Romania—19 (6.7%), and Turkey—18 (6.4%)). The distribution regarding the number of respondents was more balanced across the other 32 represented countries (Figure 1).

Out of the respondents, 166 (58.9%) were currently working in a university hospital and 72 (25.5%) in a public hospital. In addition, 28 (9.9%) were employed in a private hospital or private practice during the survey, while the remaining individuals worked in related fields [1 person (0.4%) in the pharma industry, 13 persons (4.6%) in research hospitals), or were unemployed (2 persons (0.7%)].

Of the 37 countries represented in our survey, 15 (40.5%) countries responded as not having dedicated neurosonology curricula during residency training (Albania, Armenia, Azerbaijan, Belarus, Belgium, Croatia, Cyprus, France, Georgia, Ireland, Norway, Poland, Slovenia, and the United Kingdom). On the other hand, respondents from six countries reported having dedicated neurosonology curricula (Austria, Lithuania, Montenegro, Portugal, Slovakia, and Switzerland). The existence of a dedicated training curriculum was uncertain to respondents from Denmark and Estonia.

Of the 37 countries, 14 countries saw variability within the same countries among their respondents. Germany, Greece, Hungary, Italy, Kazakhstan, Moldova, North Macedonia, Romania, Russia, Serbia, Spain, Turkey, Ukraine, and Uzbekistan were among the countries where answers showed discrepancies across different centers.

At an individual level, of the 282 respondents, 110 (38.7%) had a neurosonology training curriculum included in their residency program, while 154 of them (54.6%) did not. A total of 18 respondents (6.4%) were unsure.

Of the 110 respondents who had a dedicated neurosonology training curriculum during residency, 99 (90%) provided the duration of their training. Of them, 21 (21.21%) had less than a month for training, 63 (63.64%) had a period of training between 1 and 3 months, and 13 (13.13%) trained for 4 to 6 months. Only two persons reported training for 7 to 12 months or more than 12 months, respectively.

Of the 110 respondents that had neurosonology training in their curricula, 89 (80.9%) were trained in cervical ultrasound and Doppler, 81 (73.6%) were trained in transcranial ultrasound and Doppler, and 34 (14.2%) received training in transcranial Doppler with monitoring headframe (HITS, cerebrovascular reactivity, contrast-enhanced, etc.) as well. Twenty-seven (24.5%) of the respondents had intensive care and emergency department applications of neurosonology included in their curricula, while neuromuscular ultrasound was almost absent from the curricula across Europe, with eight (7.3%) of the respondents being trained in this area.

Looking at the bigger picture, of all the respondents, 9.6% had a residency curriculum that included intensive care and emergency department applications of neurosonology. Similarly, 12.1% had a curriculum that included transcranial Doppler with monitoring headframe, and 2.8% were taught neuromuscular ultrasound.

Finally, the median number of modules being taught was 2, with most respondents being taught cervical ultrasound and Doppler combined with transcranial ultrasound and Doppler, as reported by 79 out of 110 respondents (71.8%).

At the time of completing the survey, 141 participants (50.0%) had no experience in performing neurosonology examinations, 53 participants (18.8%) had performed a maximum of 10 examinations, 42 participants (14.9%) had performed between 11 and 60 examinations, 16 participants (5.7%) had performed between 60 and 120 examinations, while only 30 participants (10.6%) had performed over 120 examinations.

Respondents rated access to theoretical knowledge regarding neurosonology during residency as either very poor (28.0%), poor (20.2%), neutral (25.9%), good (19.3%), or very good (6.6%). Access to practical skills was also rated as very poor (30.9%), poor (18.9%), neutral (22.6%), good (18.1%), or very good (9.5%).

Overall, the respondents found that acquiring practical skills in neurosonology was extremely easy in 31 cases (12.8%), easy in 29 cases (11.9%), medium in 107 cases (44.0%), difficult in 45 cases (18.5%), and very difficult in 31 (12.8%).

The majority of neurosonology examinations in Europe are conducted by neurologists, according to 137 respondents (63.7%). Radiologists took the second position, with 43 (20.0%) of the examinations, while technicians were responsible for neurosonology examination in 33 of the responses (15.3%). Only two centers reported cardiologists as being responsible for the examinations (2 answers—0.9%).

Of the survey respondents, 91 (32.3%) stated that national certification was attainable in neurosonology in their country. In contrast, 66 (23.4%) reported that certification was unavailable, while 86 (44.3%) were unsure of its availability. National certification in neurosonology was reported as available in 20 European countries (Austria, Belarus, Georgia, Germany, Greece, Hungary, Italy, Lithuania, Moldova, Poland, Portugal, Romania, Russia, Serbia, Slovakia, Spain, Switzerland, Turkey, Ukraine, and Uzbekistan).

Of the 91 persons who responded that they were aware of a neurosonology certification in their country, 6 (6.6%) considered it very easy to obtain, 10 (11.0%) considered it easy, 47 (51.6%) saw it as medium difficulty, while 14 (15.4%) answered it was difficult, and the same numbers were recorded for very difficult.

Of the 91 survey respondents who were aware of a national certification system, 12 people (15.0%) saw no significant obstacle in getting certified. The remainder of 79 respondents all saw at least one major obstacle in the way of getting certified, with 20 (25.3%) pointing out the price of certification and 36 (45.6%) claiming difficulties in enrolling due to an insufficient number of certifying centers, while lack of practical skill gained in residency was the most frequent complaint (44 of the respondents (55.7%)). Lack of usefulness was reported by 13 (16.5%) respondents, and 3 (3.8%) considered the examination too difficult.

At the time of completing the survey, only 16 respondents (5.7%) had a national certification in neurosonology, with 10 others (3.5%) being in the process of receiving certification. A total of 256 (90.8%) of the respondents were not certified in neurosonology at the time of survey completion.

Out of the 16 respondents who were certified in neurosonology, 6 (37.5%) rated their certification experience as “medium,” 4 (25%) were “displeased” or “very displeased,” and 6 (37.5%) combined were “happy” or “extremely happy.”

Out of the 16 respondents who were certified in neurosonology, 11 (68.75%) received training in cervical ultrasound and Doppler during certification training, while 13 (81.25%) were trained in transcranial ultrasound and Doppler. In addition, 4 (25.0%) received training in neuromuscular ultrasound, 6 (37.5%) in transcranial Doppler with a monitoring headframe, and 6 (37.5%) in intensive care and ER applications of neurosonology.

The most common complaints regarding the neurosonology certification process were as follows: Prices were too high (25.0%), theoretical knowledge taught was below expectations (12.5%), there were too many students per instructor (37.5%), and the certification training module was too short (25%).

Out of the 282 respondents, 176 (62.4%) expressed interest in obtaining a European Certification in Neurosonology if that was available, while 82 (29.1%) were undecided, and 24 (8.5%) were not interested.

## Discussion

Residency programs in neurology vary greatly across Europe, with important regional differences regarding duration, mandatory external rotations, training in clinical neurophysiology, and many other aspects (11). In 2022, an update was published on the European Training Requirements in Neurology (ETRN) by EAN-UEMS, reflecting emerging requirements for the practice of neurology and contributing to the international standardization of the residency curricula. According to this consensus, neurosonology should be a part of the Neurology residency training program and preferably be covered in year 3 or 4 of training (12).

Clinical neurophysiology (including EEG, nerve conduction studies, evoked potentials, polysomnography, and neurosonology) is an area of heterogeneity at both national and international levels, with few guidelines in place regarding a standardized approach. Data are scarce, but the few articles covering the topic have shown great inequality in access to training, including in neurosonology, in both the US and Europe (7, 8). While the recent consensus cited previously tries to tackle this issue, there is still no standardized curriculum for clinical neurophysiology during neurology residency training in European countries (13).

Diagnostic ultrasound finds expanding applications in neurology, particularly in cerebrovascular evaluations. It aids in etiological assessments and hemodynamic diagnosis of brain or eye ischemia, accurately characterizing conditions such as cervical vascular atherosclerosis, dissection, vasculitis, and other rare disorders (14, 15). The technique is effective in diagnosing intracranial large vessel stenosis or occlusion, assessing collateral pathways, and detecting indirect hemodynamic signs of proximal and distal pathology.

Transcranial Doppler (TCD) stands out for its sensitivity in identifying paradoxical emboli, especially in cases with a systemic right–left shunt such as a patent foramen ovale. TCD is crucial for sickle cell disease surveillance, guiding preventive transfusion timing. In subarachnoid hemorrhage, TCD proves valuable for vasospasm monitoring and treatment adjustments (16, 17). Ultrasonography detects arteriovenous shunts, and ongoing developments include cerebral vasoregulation studies. TCD facilitates monitoring hemodynamic changes related to intracranial hypertension and diagnosing cerebral circulatory arrest. Notably, ultrasonography allows easily repeatable monitoring of evolving clinical conditions or interventions, finding more and more use in the intensive care and emergency department settings (18).

Indeed, point-of-care ultrasound (POCUS) has been adapted to the needs of the neurologists in Neuro-POCUS. This bedside investigation can be crucial as an adjunct and not a substitution for computed tomography, magnetic resonance imaging, or standard comprehensive neurosonology examination. From triage in the emergency room, control, and support during interventions, aiding in the differential diagnosis and decisions regarding further management in ambulatory neurology, to examination in the ICU/stroke unit, Neuro-POCUS is becoming an invaluable tool in managing neurological patients. However, ultrasound examination is an operator-dependent diagnostic method, relying on the examiner’s skills and experience to provide valid results (19). Meanwhile, less than 10% of residents across Europe are getting trained in this.

Neuromuscular ultrasound (NMUS) could also provide key information to neurologists, being useful in peripheral nerve blocks, steroid injections for entrapment neuropathies, and botulinum toxin injections for spasticity and dystonia. It is also utilized for determining optimal biopsy and lumbar puncture sites and is gaining traction in the intensive care setting for identifying post-operative diaphragmatic palsies. In addition, NMUS is increasingly used for surgical guidance, such as identifying normal neural tissue in the resection of nerve sheath tumors and many others (20).

While NMUS is becoming an area of increased interest for neurologists, costs for laboratory equipment as well as difficulties in accessing an adequate teaching experience are some of the key barriers to a wider implementation (21). At present, the availability of apprenticeships in NMUS is a global problem, with no standard teaching structure or curriculum in place.

Our survey focuses on particularities about the neurosonology curricula in each country, types of neurosonology modules being taught across Europe, differences between access to theoretical knowledge and practical training in neurosonology, challenges to becoming certified in neurosonology, and potential interest in European/International certification.

The survey achieved a good reach, spanning 37 European countries and gathering 282 individual responses. The target population was reached, with most responses coming from residents in neurology (50%) and junior neurologists (34.0%) working in university hospitals (58.9%) or public hospitals (25.5%).

An interesting aspect was that only 6 countries reported having dedicated neurosonology training during residency, while most others either had none (15 countries) or showed discrepancies between respondents (16 countries), suggesting some centers are not providing such training while some are, and perhaps uncertainties regarding the structure of the neurology residency curricula. This reflects the current situation in residency programs across Europe, where neurosonology training seems to be mostly a matter of luck and personal interest rather than an established part of the curricula.

For the 110 responders that had a mandatory module of neurosonology training during their residency, most had either 1 to 3 months (63.6%) or less than 1 month (21.2%) to gain practical skills in neurosonology. This leads to a very low number of performed examinations, with half of the respondents having never performed a neurosonological examination, and only 10.6% of them having performed over 120 examinations. Many young neurologists feel underprepared regarding theoretical knowledge and practical skills training gained during residency, all the while more than 75.3% of respondents consider that gaining practical skills in neurosonology is a medium difficulty to a very hard task.

Consensus among experts varies, but most countries require a minimum of 100 examinations before doctors may apply for neurosonology certification, and some experts recommend as many as 1,000 examinations before having confidence in the results offered by this means of investigation (22). This may explain why most respondents across Europe seem to believe they are getting insufficiently trained in neurosonology.

This is happening despite abundant data showing the uses of neurosonology, and the need for operators ready to perform this investigation is only expected to grow in the future (19, 23, 24).

Our survey found that only 7.3% of respondents who had a neurosonology module during residency had received training in NMUS, and only 25.0% of the respondents certified in neurosonology had been trained in NMUS during their certification process. Less than 3% of residents across Europe are being taught about this branch of neurosonology.

There is a need to better prepare neurologists for neurosonology examinations as they are the overwhelming majority (63.7% in our survey) that will be performing this investigation.

National and international certifications are an important step in standardizing the pathway to better training and coverage. However, only 32.3% of our respondents were aware of a national certification in neurosonology being available in their country, and respondents from these countries saw neurosonology certification as medium to very difficult to obtain in 82.4% of cases. Price of certification, an insufficient number of certifying centers, and lack of practical skills gained in residency were the most frequent complaints.

Respondents showed a major interest in European Certification in Neurosonology, with 176 (62.4%) saying they would be interested in attaining such a certification. International certification has been available since 2022 through the European Society of Neurosonology and Cerebral Hemodynamics (ESNCH), offering two levels of certification: practical level and teaching level, based on the results obtained during testing.

A recent survey on the topic of neurosonology was published by Claudio Baracchini et al. on behalf of the Council of Nations of the European Society of Neurosonology and Cerebral Hemodynamics (ESNCH). While this survey is not focused on neurosonology training, and the respondents are national experts in the field rather than young neurologists, the authors mention that in most of the countries surveyed (69.1%), neurosonology is not part of the medical program, while in most countries (71.4%), neurosonology is part of the neurology residency training program and is mandatory in almost half (47.6%) (7). This is in contrast with our data, and the differences cannot be explained by the differences in countries included in the survey alone.

While no simple solution can be easily identified, we believe that there are sufficient arguments at this point to consider that neurosonology should be a core part of the neurology training curricula rather than an optional supra-specialization. The European Society of Neurosonology and Cerebral Hemodynamics (ESNCH), together with the European Academy of Neurology (EAN), could help unite these efforts across Europe. Representatives of the national neurology societies within the EAN can be important allies in pushing this message to relevant local authorities. The Neurosonology Scientific Panel of the EAN can be tasked with coordinating a joint effort to define a Neurosonology Curriculum for Europe, including a proposed length, number of examinations, and types of examinations to be covered. A position paper on this topic could then act as a standard that European countries should adhere to.

Our study has some important limitations. First, it includes only 282 respondents. While no official data can be easily found regarding the total number of neurologists in training currently in Europe, it is most likely in the thousands, and thus, the survey covers only a small fraction of the total possible answers. However, it is worth noting that among the responders, there are the national RRFS representatives, people who, by nature of the position, possess expert knowledge regarding the training curricula in their countries and the challenges that junior neurologists may face. Because 58.2% of the responders were from only 5 countries, this might lead to an overrepresentation of some countries, and the results might be less representative of Europe in general. Another limitation is that the survey was mainly distributed by (although not limited to) the RRFS networks, which may lead to an overrepresentation of RRFS members’ views and an underrepresentation of other junior neurologists who may not be a part of this network. Finally, by the methodology in which this survey was conducted, the presented results can only be interpreted as personal opinions of the responders rather than official national data.

## Conclusion

In conclusion, this survey sheds light on the challenges and disparities in neurosonology training and certification across Europe. The findings reveal significant variations in the inclusion of neurosonology curricula in neurology residency programs, with only a handful of countries reporting dedicated training. The limited duration of training and a lack of exposure to different neurosonology modules contribute to a low number of performed examinations by respondents, leading to difficulties in getting certified.

The survey highlights dissatisfaction among respondents regarding both theoretical knowledge and practical skills training during residency. Despite the increasing importance of neurosonology in various neurological applications, including cerebrovascular evaluations and point-of-care ultrasound (POCUS), most neurologists seem insufficiently trained in this field.

Furthermore, the survey underscores the need for standardized certification processes to ensure consistent and high-quality neurosonology practice. The current landscape indicates that many neurologists are unaware of national certification options, and those who are familiar with certification face challenges such as high costs and limited certifying centers. Due to inadequate access to training, many also report a perceived lack of practical skills gained during residency.

Interestingly, there is a strong interest among respondents in obtaining a European certification in neurosonology, with a significant majority expressing interest in this initiative. The availability of international certification through the European Society of Neurosonology and Cerebral Hemodynamics (ESNCH) offers a potential solution.

## Data Availability

The raw data supporting the conclusions of this article will be made available by the authors, without undue reservation.
