# Supplementary material for: Perceived discrepancies in neurosonology training and certification across Europe: a RRFS/EAN survey
Source: Front Neurol. 2024 Oct 28;15:1464946. doi: 10.3389/fneur.2024.1464946 (PMC11556350; doi:10.3389/fneur.2024.1464946)
Supplement: Supplementary file 1 [file Data_Sheet_1.PDF]

Survey – Neurosonology, Pain and Dementia

Thank you for your engagement in our survey.

We would like to know about educational opportunities in pain, dementia and neurosonology in European countries and how different they are.

This study aims to identify gaps and differences in education in these areas of neurology.

The survey has a general part and three separate parts for each specialty. It will take you up to **15 minutes**.

General questions

Q1
What is your current training level? (multiple answers possible)

- Junior neurologist/consultant
- PostDoc
- Resident (in training)
- PhD student
- Other (please specify)

Q2
In what country will most of your Neurology training take place / has taken place? (all further questions related to "your country" will refer to this as well)

- A dropdown list of all European countries

Q3
Do you work in a:

- University hospital
- Research hospital
- Public hospital
- Private hospital

- Private practice
- Other (please specify)

Neurosonology

1.
Does the Neurology Residency curriculum in your country include a dedicated time for Neurosonology training?

- Yes
- No
- I don’t know

If yes – proceed to question 2
If not/don’t know – jump to question 4

2
How much mandatory time is allocated for Neurosonology training during residency in your country?

- < 1 month
- 1-3 months
- 4-6 months
- 7-12 months
- > 12 months

3
What applications of Neurosonology were you taught during your Residency training?

- Cervical ultrasound and Doppler
- Transcranial ultrasound and Doppler
- Neuromuscular ultrasound
- Transcranial Doppler with monitoring headframe (HITS, cerebrovascular reactivity, contrast-enhanced, etc.)
- Intensive Care and Emergency Department applications of Neurosonology
- Other (please specify)

4
During your residency, how would you rate the access to the theoretical knowledge regarding Neurosonology provided by your training centre? (1=very poor; 2= poor; 3=neutral; 4= good; 5=very good)

- Scale of 1-5

5
During your residency, how would you rate the access to practical training in Neurosonology provided by your training center? (1=very poor; 2= poor; 3=neutral; 4= good; 5=very good)

- Scale of 1-5

6
How many Neurosonology examinations have you accumulated during your residency?

- No practical experience in examinations
- Between 1 to 10 examinations
- Between 11 to 60 examinations
- Between 61 to 120 examinations
- Over 120 examinations

7

What is your perception of gaining practical skills in Neurosonology?
Scale of 1-5 (1 - extremely easy, 5 - extremely difficult)

8
Who performs the Neurosonology examination in your hospital?

- The Neurologist
- The technician
- The radiologist
- The cardiologist
- Other specialities

9
Is there a National certification in Neurosonology attainable in your country?

- Yes
- No
- I don’t know

If yes – proceed to question 10
If not/don’t know – jump to question 12

10
I perceive the opportunity to be certified in Neurosonology in my country as…. (1 = extremely easy, 5=extremely difficult)

- Scale of 1-5

11
What are the main challenges in becoming certified in Neurosonology in your country?

- Price of certification
- The number of certification centres is too limited compared to the demand (challenging to enrol for certification)
- Lack of practical skills gained during Residency
- The lack of usefulness once certification is attained (no place to perform Neurosonology on the current job)
- The certification examination is too difficult.
- I don't think there are any significant obstacles to Neurosonology certification.
- Others (Please specify)

12
Do you have a National Certification in Neurosonology when completing this survey?

- Yes, obtained in my country
- Yes, obtained in any other country
- No
- No, but I am currently in the process of receiving certification.

If yes or currently under certification– proceed to question 13
If not – jump to question 16

13
On a scale from 1 to 5, how satisfied were you with your National Certification in Neurosonology experience? (1=very displeased, 5=extremely happy with it)
Scale of 1-5

14
What applications of Neurosonology were you taught during your Neurosonology Certification Training?

- Cervical ultrasound and Doppler
- Transcranial ultrasound and Doppler
- Neuromuscular ultrasound
- Transcranial Doppler with monitoring headframe (HITS, cerebrovascular reactivity, contrast-enhanced, etc.)
- Intensive Care and Emergency Department applications of Neurosonology
- Other (Please specify)

15. What was your experience regarding the national certification process?

- The certification price was adequate compared to the quality of the classes.
- The certification price was too high compared to the quality of the classes.
- The level of theoretical knowledge that was taught during classes was beyond my expectations.
- The level of theoretical knowledge that was taught during classes was under my expectations.
- The workshops (practical skills sessions) were beyond my expectations
- The workshops (practical skills sessions) were under my expectations
- The amount of student was adequate for the number of available teachers/instructors
- There were too many students for the number of available teachers/instructors
- The duration of the certification module is too long
- The duration of the certification module is too short
- Other (Please specify)

16. If there was a European Neurosonology Certification, would you be interested in becoming certified?

- Yes
- No
- Undecided

This survey also contained 2 other sections, regarding Pain and Dementia.
The full survey can be available on request to the corresponding author.
